# Pre-vaccine era cervical human papillomavirus infection among screening population of women in west Austria

**DOI:** 10.1186/s12889-016-3581-0

**Published:** 2016-08-26

**Authors:** Wegene Borena, Margarethe Grünberger, Andreas Widschwendter, Karl Heinz Kraxner, Elisabeth Marth, Peter Mayr, Joerg Meier, Norman Ruth, Aida Tort Guerrero, Christian Marth, Dorothee Holm-von Laer

**Affiliations:** 1 Division of Virology, Medical University of Innsbruck, Schoepfstrasse 41, 6020 Innsbruck, Austria; 2 Department of Gynaecology and Obstetrics, Medical University of Innsbruck, Innsbruck, Austria; 3 Landeck, Austria; 4 Innsbruck, Austria; 5 Kufstein, Austria; 6 Imst, Austria; 7 Woergl, Austria

**Keywords:** HPV epidemiology, Womens’ health, Cervical cancer, HPV vaccine

## Abstract

**Background:**

In order to evaluate the newly implemented gender-neutral HPV vaccination program, knowledge on the pre-vaccine prevalence of HPV infection is of paramount importance. Data on HPV infection among the women with no known previous cytological abnormalities are inexistent in Austria. This study presents data on the prevalence and distribution of HPV genotypes among women with no known cytological abnormalities in west Austria.

**Methods:**

Women between 18 and 65 years of age attending annual cervical cancer screening examinations were included in the study. Data on socio-demographic and reproductive factors were collected using structured questionnaires. Corresponding cervical swab samples were tested for the presence of HPV DNA and were genotyped. Questionnaire data and HPV status were linked with the corresponding cytological findings.

**Results:**

A total of 542 women were included in the study. The mean age of the study participants was 35.9 (SD = 11.5). The prevalence of HPV infection was 20.5 %. HPV 16 (6.5 %), HPV 33 (3.3 %) and HPV 31 (3.0 %) were the dominant genotypes detected. Multivariate analysis showed that women younger than 30 years of age, smokers, women with a higher number of lifetime sexual partners and those living in the eastern districts of the study region were at significantly higher risk of HPV infection.

**Conclusions:**

With this study we present the first data on the prevalence of cervical HPV genotypes among a screening population in Austria. The results not only fill the missing information on HPV infection in this group of women in the country, they also provide baseline data for a future evaluation of the impact of the Austrian gender-neutral HPV immunization program. Moreover, our finding of higher HPV prevalence in the eastern compared to the western district of the study region may – at least partly – explain the east–west gradient in the standardized incidence rate of cervical cancer in the region.

**Electronic supplementary material:**

The online version of this article (doi:10.1186/s12889-016-3581-0) contains supplementary material, which is available to authorized users.

## Background

Genital human papillomavirus (HPV) infection is the most common sexually transmitted viral infection [1]. It is mostly transient resulting in no cervical lesions or leading to low-grade lesions that often regress spontaneously. However, a non-negligible proportion of the infections persists and results in subsequent pre-cancerous and cancerous lesions [2, 3].

For the prevention of cervical and other genital neoplasia, three vaccines – Gardasil® *(quadrivalent vaccine against HPV types 6, 11, 16 and 18),* Gardasil 9® *(nonavalent vaccine against HPV types 6, 11, 16, 18, 31, 33, 45, 52 and 58)* and Cervarix® *(bivalent vaccine against HPV types 16 and 18)* – have been approved for use [4–7]. Developed nations like Australia and several countries in Europe and North America have integrated HPV immunisation in their vaccination programs soon after the approval. Accordingly, in these countries significant reductions have been observed in many high-risk HPV (hrHPV) infections as well as in HPV-associated warts and pre-cancerous lesion [8–10].

In Austria these vaccines have been recommended and available for use since their approval [11, 12]. However, it is only since the beginning of 2014 that the country integrated HPV vaccine in the free of charge immunization program [12]. Starting from the autumn of 2014, 4th grade elementary school girls and boys receive two doses of Gardasil (0–6 months) as part of school-based immunization program. In order to assess the long-term impact of this program, pre-vaccine data on the prevalence of HPV infection and genotype distribution are of paramount importance [11].

Data on the prevalence of HPV infection is scarce in Austria. Until the end of 2013 the only data available were those on HPV prevalence in cervical cancer samples [13, 14]. L Rössler et al. recently published data on the prevalence of HPV among Austrian women with high- grade intraepithelial lesions [15]. However, to date, there are no data on the epidemiology of HPV infection among women without any known cytological abnormalities (Additional file 1: Figure S1). One of the early parameters in evaluating the success of HPV immunisation programs is the comparison of HPV prevalence in the pre- and post-HPV-vaccine era.

The main aim of this study is to present data on the distribution of HPV genotypes among screening population of women in west Austria. With this study, we also aim to address the role of HPV infection in an interesting difference in the cervical cancer incidence observed between eastern (standardized incidence rate (SIR) in the range of 1.2 to 1.5) and western (SIR in the range of 0.6 to 0.9) districts of the study region [16]. To our knowledge this is the first report on the epidemiology of cervical HPV infection among screening population of women in Austria.

## Methods

### Study population

The study population included sexually active women aged 18–65 years undergoing routine gynaecological screening at five randomly selected gynaecological clinics in west Austria (Tyrol) between March 2013 and February 2015. Screening for cervical cancer in Austria is opportunistic in nature but is provided free-of-charge at a yearly basis. Women who have consented to participate in the study filled out questionnaires on socio-demographic, reproductive and sexual characteristics. The questionnaires were then sent back to our institute (Division of Virology of the Medical University of Innsbruck) with the corresponding cervical samples.

For the ascertainment of persistent HPV infection, those women who tested positive for one or more of the known hrHPV types were invited for a follow up HPV testing approximately a year after the baseline.

### The samples

One of the two swabs taken from the patient was sent to our institute, the other one was sent to a pathology laboratory (Dr. P. O.) for cytological (PAP) examination. Cervical swabs were taken with Abbott Cervi-Collect specimen collection kit. DNA extraction (Biomerieux EasyMag 2.0) took place within 2 days of arrival of samples at our institute. The extracted DNA samples were stored at −20 °C until HPV testing.

### Detection and Genotyping of HPV

For the detection and genotyping of HPV, we used a two-step testing procedure. The first test is a real-time PCR that identifies the presence or absence of HPV DNA (HPV RealQuality, AB Analitica, Padova, Italy). The second step involves genotyping of all positive samples using a reverse line blot hybridization system (AmpliQuality HPV-TYPE EXPRESS, AB Analitica, Padova, Italy). This genotyping kit was able to identify 40 different HPV types, including all high-risk, probable high-risk and low-risk types. Membrane stripes coated with genotype specific DNA probes are used to identify the different genotypes through hybridization of denatured PCR product. Multiple infections were easily detected. The analysis includes dUTP/UNG system for the prevention of contamination due to carry-over.

### Cytology

Cytological examinations were interpreted according to Munich Nomenclature for the Cytological Diagnosis of Cervical Pap smears [17]. In our study cytological findings were classified as abnormal (PAP III+) if the pathologist reported PAP III (unclear finding corresponding in the Bethesda classification system to ASCUS), PAP IIID (cells of mild or moderate dysplasia corresponding in the Bethesda classification system to LSIL or HSIL respectively), PAP IVA (cells of severe dysplasia or carcinoma in situ corresponding in the Bethesda classification system to HSIL or AIS, respectively), PAP IVB (cells of invasive carcinoma not safely excluded, corresponding in the Bethesda classification system to HSIL or AIS with features suspicious for invasion, respectively) or PAP V (cells of invasive cervical carcinoma).

### Data analysis

HPV prevalence was computed in crude and age standardized manner. Chi square or Fischer’s exact test (two-tailed) were used for analysis of distribution of several hrHPV genotypes. Logistic regression model was used to compute odds ratios for HPV positivity across several variables including smoking status, educational level, marital status, number of life time sexual partners and age at first sexual contact with corresponding 95 % confidence intervals. *P*-values <0.05 were considered significant. Prevalence ratio of HPV infection was also computed across Tyrolean districts.

Statistical analyses were performed in SPSS (Version 20.0. Armonk, NY: IBM Corp.).

## Results

### Baseline characteristics

Baseline characteristics of the study participants are presented in Table 1. A total of 542 women were included in this study. The mean age of the study participants was 35.9 (SD = 11.5) years. One third of the women were current smokers and 27 % were overweight. Mean age at first sexual contact was 16.8 years (SD = 2.2). A quarter of the study participants reported to have had more than five life-time sexual partners. Two hundred thirteen (39.3 %) participants in this study were from eastern Tyrolean districts (Kufstein, Kitzbühel) whereas 205 (37.8 %) represent two western districts (Imst and Landeck). Just over a fifth of the participants were from the city of Innsbruck (IBK) or its suburbs (IL). Additional file 2: Figure S2 presents a map of the studied region.

**Table 1:** Baseline characteristics of study participants

	Total (*n* = 542)
Age, years	
Mean (SD)	35.9 (11.5)
Median	34.5
Participants per district, *n* (%)	
Oberland	205 (37.8)
Unterland	213 (39.3)
IBK	68 (12.5)
IL and others	55 (10.1)
BMI, kg/m2	
Mean (SD)	23.4 (4.0)
Smoking status, *n* (%)	
Current smoker	175 (32.3)
Non-smokers	364 (67.1)
Life time sexual Partners, *n* (%)	
≤ 5	340 (62.7)
6–10	95 (17.5)
≥ 11	41 (7.6)
HPV vaccinated, *n* (%)	
At least one vaccine	20 (3.7)
Reasons for not being vaccinated, *n* (%)	
Did not know	201 (37.1)
Expensive	42 (7.8)
Fear of side effect	78 (14.4)
Other reasons	155 (28.6)
No response	86 (15.9)
If vaccine available for free, *n* (%)	
Will get vaccinated	179 (33)
Will not get vaccinated	72 (13.3)
Do not know	233 (43)
No response	58 (10.7)

*SD* standard deviation, *BMI* body mass index

*Oberland* western districts (Imst, Landeck)

*Unterland* Eastern districts (Kufstein, Kitzbuehel), *IBK* Innsbruck, *IL* suburbs of Innsbruck

### HPV immunization

Immunisation coverage among the studied population was very low (3.7 %). The attitude of the study participants towards HPV vaccine is presented in Table 1. Not knowing about the vaccine and/or not being informed about it was the most frequent reason given by the study participants for not being vaccinated. When asked if they would get vaccinated provided the vaccine was available for free, the majority of the study participants showed undecidedness and a minority (13 %) declined clearly.

### HPV prevalence

**Fig. 1:**
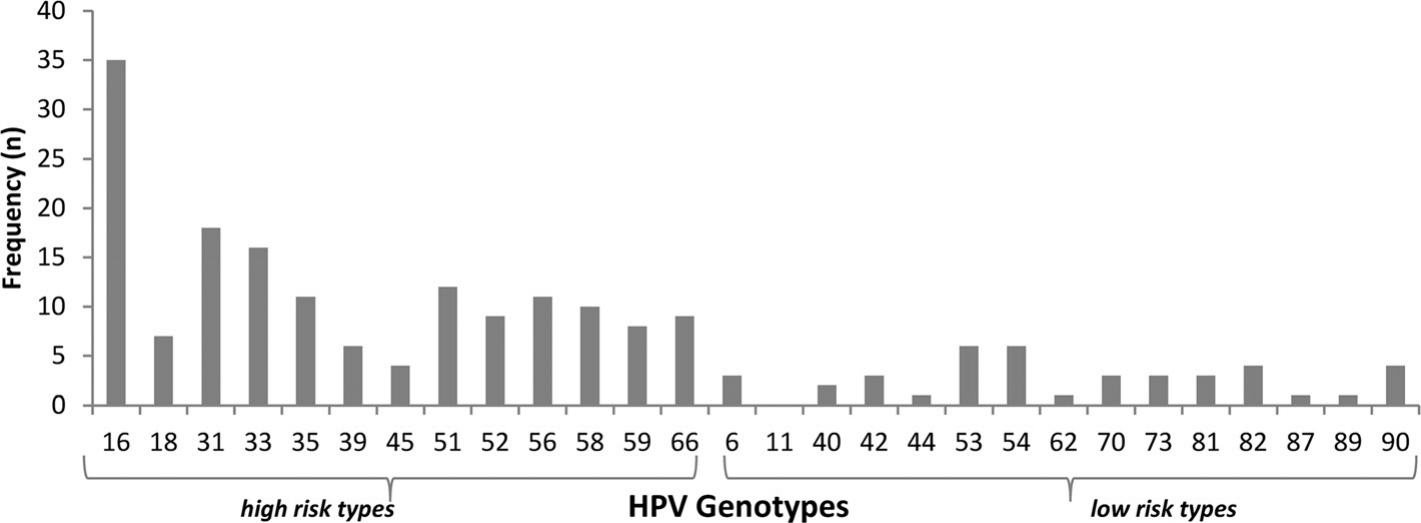
Distribution (%) of all detected HPV genotypes among screening population of women in west Austria

Figure 1 shows the distribution of all detected genotypes among HPV positive women. A total of 111 (20.5 %) participants were positive for one or more of HPV genotypes. Among the high risk types HPV 16 is the most commonly detected genotype followed by HPV 33 and HPV 31. Out of the low risk genotypes HPV 53 and HPV 54 are the most commonly detected types followed by HPVs 82 and 90. Only three participants (2.7 % of HPV positive participants) were positive for HPV 6 and none had HPV 11.

Fifty women (45.5 %) had HPV infection with multiple genotypes whereas the slight majority presented with single-genotype infections (Additional file 3: Figure S3). The majority of the participants (91.8 %) are positive for one of the genotypes classified as definitive, probable or possible carcinogenic HPV types (IARC classification) (Additional file 4: Figure S4) whereas only nine of HPV positive participants (8.2 %) had infections with only low risk genotypes [18]. A total of 73 women (65.7 % of HPV positive women) are positive for one or more of the high-risk genotypes that are covered in the nonavalent HPV vaccine (HPV-types 16, 18, 31, 33, 45, 52 and 58).

Table 2 characterises the prevalence of any HPV and selected high-risk types by socio-domographic and behavioural data. HPV infection was significantly higher among individuals younger than 30 years of age. Smoking, being single and/or divorced, having several lifetime sexual partners and starting sexual contact at a younger age were significantly associated with higher cervical HPV DNA detection rate. On the contrary higher body mass index (BMI) was not associated with increased risk of HPV infection showing rather a protective effect with a borderline statistical significance. Analysis across Tyrolean districts showed that HPV prevalence was higher among women from the eastern districts compared to those from the west. The significant association disappeared when analysis was adjusted for age, indicating that the observed difference may be due to differences in the age distribution of the study participants in these two regions. A further analysis was done among women above the age of 30 years (*n* = 342) as the clinical and diagnostic relevance of HPV infection is higher in women beyond the age of 30 (Table 3). In this sub-population we found no significant difference in the age distribution across the districts (*P* = 0.53). Multivariable analysis of HPV prevalence in this age group showed still significantly higher HPV prevalence in the eastern compared to the western districts.

**Table 2:** Prevalence of HPV Infection by demographic and behavioural characteristics among non-vaccinated women (*n* = 522)^a^

Variables	Variable categories	Any HPV type	HrHPV^b^
		(*n* = 111)	(*n* = 73)
		OR (95 % CI)	OR (95 % CI)
Age	<30 years	1	1
	≥30 years	0.38 (0.25–0.60)*	0.34 (0.21–0.57)*
Marital status	Married/Partnershaft	1	1
	Single/divorced	1.87 (1.17–2.99)*,^e^	1.16 (0.66–2.10)
Educational status	Basic schooling	1	1
	High school and more	0.81 (0.53–1.25)	0.83 (0.50–1.37)
Districts	Oberland^c^	1	1
	Unterland^d^	1.76 (1.06–2.92)*	1.57 (0.88–2.79)
Smoking	non-smoker	1	1
	smoker	4.10 (2.6–6.41)*	3.16 (1.89–5.26)*
BMI	<25	1	1
	≥25	0.55 (0.32–0.95)*	0.64 (0.34–1.19)
Age at first sexual contact	<16 years	1	1
	≥16 years	0.44 (0.27–0.72)*	0.61 (0.35–1.08)
LSP	<6	1	1
	≥6	2.40 (1.39–3.62)*	1.95 (1.13–3.36)*

*BMI* body mass index, *LSP* number of life time sexual partners, *OR* odds ratio, *CI* confidence interval

*p-value = statistically significant

^a^only non-vaccinated women

^b^HPVs 16, 18, 31, 33, 45, 52 58 (all the HrHPV types included in nonavalen HPV vaccine)

^c^western districts (Imst, Landeck)

^d^Eastern districts (Kufstein, Kitzbuehel)

^e^significant for study participants above 30 years of age

**Table 3:** Multivariable adjusted prevalence of HPV Infection by demographic and behavioural characteristics among women above and below 30 years of age^a^

		Women ≥30 years		Women < 30 years	
Variables	Variable categories	Any HPV type	HrHPV^b^	Any HPV type	HrHPV^b^
		(*n* = 49)	(*n* = 31)	(*n* = 55)	(*n* = 41)
		OR (95 % CI)	OR (95 % CI)	OR (95 % CI)^a^	OR (95 % CI)
Marital status	Married/Partnershaft	1	1	1	1
	Single/divorced	1.89 (0.87–4.14)	1.28 (0.49–3.32)	1.29 (0.58–2.85)	0.82 (0.35–1.93)
Educational status	Basic schooling	1	1	1	1
	High school and more	0.96 (0.47–1.97)	1.60 (0.67–3.82)	0.52 (0.24–1.17)	0.39 (0.17–0.94)*
Districts	Oberland^c^	1	1	1	1
	Unterland^d^	2.43 (1.06–5.60)*	2.66 (0.98–7.24)^e^	0.79 (0.32–1.96)	0.98 (0.38–2.50)
Smoking	non-smoker	1	1	1	1
	smoker	2.64 (1.30–5.37)*	2.09 (0.90–4.84)	3.34 (1.53–7.29)*	3.07 (1.33–7.09)*
BMI	<25	1	1	1	1
	≥25	0.39 (0.16–0.96)*	0.38 (0.13–1.17)	1.30 (0.51–3.30)	1.98 (0.84–4.67)
Age at first sexual contact	<16 years	1	1	1	1
	≥16 years	1.06 (0.37–3.00)	2.43 (0.51–11.5)	0.88 (0.40–1.94)	1.18 (0.50–2.77)
LSP	<6	1	1	1	1
	≥6	2.23 (1.06–4.68)*	2.11 (0.88–5.06)	0.86 (0.39–1.88)	0.98 (0.43–2.71)

*BMI* body mass index, *LSP* number of life time sexual partners

*OR* odds ratio, *CI* confidence interval, *LSP* lifetime sexual partner

*p-value = statistically significant

^a^analysis using only non-vaccinated women, analysis adjusted for age, age at first sexual contact, LSP and smoking

^b^HPVs 16, 18, 31, 33, 45, 52 58 (all the HrHPV types included in nonavalen HPV vaccine)

^c^western districts (Imst, Landeck)

^d^Eastern districts (Kufstein, Kitzbuehel)

^e^borderline significant

**Fig. 2:**
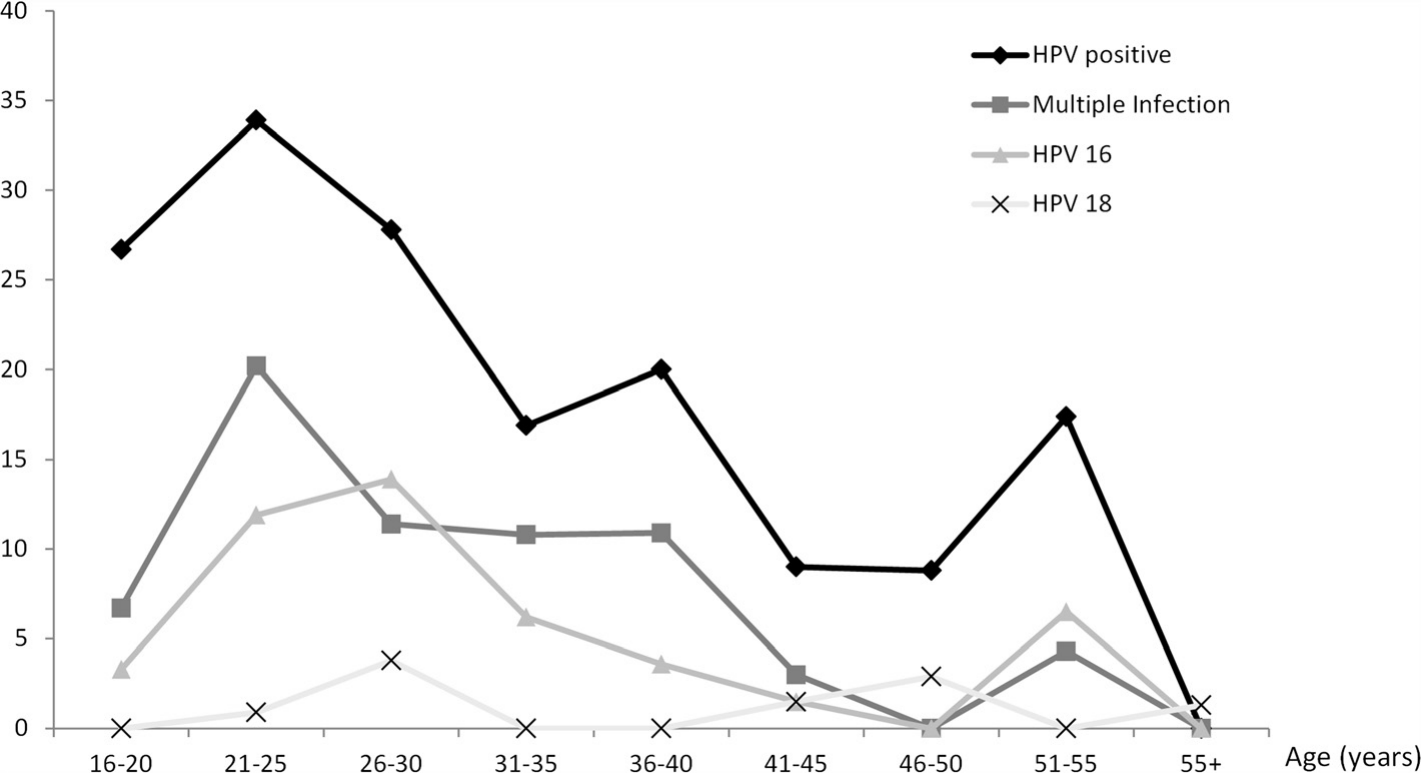
Trends in age specific HPV prevalence among screening population of women in west Austria

The peak age of overall HPV infection, multiple infections and infection with the commonest genotypes lies between 21 and 25 years. This is followed by two lower peaks at 36 to 40 years and at 50 to 55 years. Figure 2 shows the trends in HPV prevalence across age groups. The overall trends in HPV prevalence peaks were statistically significant for overall HPV positivity (*P* = 0.001) and for common high risk type infections (*P* = 0.035).

The compliance for HPV re-testing a year after the initial test was low. A total of only 48 (47.1 %) women initially positive for any hrHPV genotype were re-tested 1 year after the baseline examination. Twenty five (52.1 %) of the infections were persistent. No statistically significant association was observed between HPV persistence and any of the sociodemographic factors.

### Cytology

Data on cytological examination were available for a total of 384 women (Table 4). Twenty one (3.7 %) women were diagnosed with PAPIII+, which were significantly associated with overall, high risk type and multiple HPV infection (P < 0.0001). Of the high-risk types, HPV 16 and 18 were significantly associated with adverse cytological outcomes. Twenty four percent of HPV 16 positive women have accompanying cytological findings of PAP III+. The proportion of smokers is significantly higher among those with PAP III+ [OR = 4.15 (95 % CI: 1.63–10.57)]. A total of 48 high-risk positive patients were re-tested once again 9–15 months after the baseline examination. Twenty-three (52 %) women had persistent infection, the majority of them being HPV 16. Persistent HPV infection was higher among those with abnormal PAP finding, however not statistically significant [OR = 1.56 (95 % CI: 0.41–5.84)].

**Table 4:** HPV prevalence and distribution of high risk and low risk genotypes across cytological findings

	PAP positive	PAP negative
	(PAP III+) (*n* = 21)	(PAP I-II) (*n* = 363)
Age, years	32.6 (8.1)	35.9 (11.5)
Mean (SD)		
Age at firts sexual contact, years	16.8 (2.8)	16.8 (2.4)
Mean (SD)		
BMI, Mean (SD)	22.5 (3.3)	23.4 (4.0)
Current smoker, *n* (%)	14 (66.7)	117 (32.5)*
HPV positive, *n* (%)	19 (90.5)	75 (20.7)*
Multiple HPV infection, *n* (%)	7 (33.3)	33 (9.1)*
Nonavalent type HrHPV, *n* (%)^a^	15 (71.4)	48 (13.2)*
HPV 16, *n* (%)	9 (45)	20 (5.8)*
HPV 18, *n* (%)	2 (9.5)	4 (1.1)*
HPV 31, *n* (%)	1 (4.8)	13 (3.6)
HPV 33, *n* (%)	1 (4.8)	14 (3.9)

*SD* standard deviation, *HrHPV* high risk HPV genotypes

*PAP III +* abnormal PAPanicolaou (cervical smear cytology) including PAP III, PAP IIID, PAP IVA and IVB and PAP V

*PAPI-II* normal PAPanicolaou (cervical smear cytology)

*P = sig

^a^16, 18, 31, 33, 45, 52 58 (all HrHPV types included in nonavalen HPV vaccine)

## Discussion

### HPV prevalence

Prevalence of HPV DNA in a screening population of women aged 18 to 65 years in west Austria was 20.5 %, with the highest prevalence (33.9 %) seen among women aged 21 to 25 years. Although the overall prevalence was slightly higher than the crude HPV prevalence worldwide, the pattern of age specific HPV prevalence was shown to correspond to that seen in other regions [19–21]. Slight differences may be due to differences in the type of test used for the HPV detection or differences in the study population selected. Slightly over 60 % of all the infections are detected among women below 25 years of age, which may represent a high susceptibility of HPV infection right after sexual debut. Like in studies from other region, our study also showed other smaller peaks in higher age groups [22]. There is no clear reason as to why HPV prevalence peaks once again in a later age. One commonly accepted assumption is the fact that divorce and separation tends to put many women back into new-partner sexual relationships. Previous studies attribute the increased prevalence of sexually transmitted infections among older women to an increased divorce rate [23, 24]. Demographic data in Austria (Statistik Austria) report that the peak age for divorce is around 35–40 years [24] which corresponds to our finding of a second HPV peak at around this age range. The second HPV re-peak between 51 and 55 years of age may likewise be explained by an increased risk of new infections as well as by the accompanying age-related anatomical changes in the vulva and vaginal mucosa following menopause at this age. Another explanation for the HPV surge in an older age may be the hypothesis of a latent HPV infection persisting below detection limit and reactivating at some point due to declining immunity or other comorbidities commonly seen in advanced age. This hypothesis has been evidenced in some animal studies or in patients with recurrent respiratory papillomatosis [25–28].

As in several other studies, HPV 16 is clearly the most dominant genotype. It is detected in cervical samples of one out of 20 of the study population. Although HPV 18 is the second common genotype present in cervical cancer samples, it is obviously surpassed by several other genotypes among women with no known cytological abnormalities [29]. Genotypes 31 and 33 – for example - were shown to dominate cervical infection following HPV 16 both in our study and in a study from several European countries [29]. This pattern was also shown to persist among women with high-grade intraepithelial neoplasia [29]. Moreover, data from the WHO ICO information centre on HPV infection (last updated on 20th March 2015) reports that HPV 33 may be the second common genotype detected in cervical cancer samples next to HPV 16 in Austria [30]. The plausibility of this report, however, is questionable. Moreover, in our study, only one of the women with HPV 33 positive samples was diagnosed with abnormal PAP. Further epidemiological studies need to be undertaken in order to clarify or solidify this prevalence pattern among cervical cancer patients in Austria.

### HPV infection and lifestyle factors

Our study showed a threefold higher risk of HPV infection among current smokers, which persisted even after adjusting for other behavioural factors like number of lifetime sexual partners and age at first sexual contact. This might indicate that smoking per se predisposes to HPV infection as is also shown by other studies [31–33]. Tobacco use was reported to have local and systemic immunosuppression effect increasing the probability of acquisition of an HPV infection following an exposure [33]. The exact biological mechanism, however, is not yet clear. This highlights the need to evaluate the exact role of smoking in the natural history of genital HPV infection. Contrary to smoking, high BMI was not associated with increased risk of HPV infection. The fact that HPV infection was significantly higher in non-overweight individuals in our study might be a finding by chance or it might indicate a possible association between physical appearance and sexual behaviour.

### HPV infection across Tyrolean districts

Our finding of higher HPV prevalence in eastern districts of Tyrol compared to those in the west seems to parallel the regional differences in cervical cancer incidence and mortality observed by W Oberaigner et al. [16]. This association persisted even after eliminating the significant age difference between the two regions. Other confounding factors like number of life time sexual partners and age at first sexual contact were considered and adjusted for. Yet the significant difference in HPV prevalence between east and west was persistent, particularly among women above the age of 30 years. However, since questions regarding sexual behaviour may be sensitive, responders’ bias and residual confounding due to these factors can still not be convincingly excluded. Other host and /or pathogen associated factors differentially predisposing to HPV infection and persistence across these geographical regions may be interesting to investigate.

### HPV immunization

Surveys from several countries that have introduced free-of-charge HPV vaccination program since the approval of the vaccines have demonstrated a high vaccine coverage rate of more than 70 % [8, 34]. Accordingly, the prevalence of hrHPV infection has reduced significantly in the years after vaccine introduction. The low immunization coverage (3.7 %) in the Austrian population may be, on one hand, a reflection of the reluctance of the country to provide the vaccine free of charge for the eligible age group coupled with the conservative view of the population towards vaccines particularly towards newly introduced vaccines. On the other hand, however, with the median age of 35 years, the majority of our study population was beyond the age of eligibility as the vaccines were introduced explaining the low coverage to some extent. Our study assessed the attitudes of the study population towards HPV vaccine. The fact that majority of the studied women showed undecidedness about getting vaccinated- which may indirectly reflect undecidedness about letting their children vaccinated - indicates the urgent demand for public health action towards raising awareness on HPV vaccine. Further surveys need to be undertaken which investigate the level of information and the possible barriers to HPV vaccine acceptance.

## Conclusion

This study presents the first data on prevalence and genotype distribution of cervical HPV infections among women with no previously known cytological abnormalities in west Austria. Factors including smoking, number of life time sexual partners, age at first sexual contact and several socio-demographic factors, namely marital status and geographical region were identified as risk factors for age adjusted HPV prevalence. The finding of higher HPV prevalence in the eastern compared to the western district of the study region may - at least partly - explain the east–west gradient in the standardized incidence rate of cervical cancer in the region. The strikingly low HPV vaccine coverage may change with the recently introduced school-based vaccination program [35]. On the other hand, this data – with the extremely low vaccine coverage rate – will serve as baseline pre-vaccine information for further evaluation of the impact of the newly implemented gender-neutral HPV vaccine program in Austria.

## Additional files

Additional file 1: Figure S1. Prevalence and distribution of ten most frequent HPV genotypes among women with no or low grade cervical lesions in Austria. (http://www.hpvcentre.net/statistics/reports/AUT_FS.pdf). Accessed March 2016. (TIF 48 kb) Additional file 2: Figure S2. Map of Tyrol, West Austria: Districts included in the study (circled) (Tyrolean regional government website. https://www.tirol.gv.at/fileadmin/themen/landesentwicklung/raumordnung/bilder/tiris/bezirksuebersicht_tirol_01.jpg. Accessed September 2015. (TIF 167 kb) Additional file 3: Figure S3. Distribution of single and multiple HPV genotypes among screening population of women in west Austria (n=542). (JPG 52 kb) Additional file 4: Figure S4. Distribution (%) of HPV genotypes (high risk types, HPV 6 and HPV 11) among screening population of women in west Austria. (TIF 221 kb)

## Abbreviations

AIS: Adenocarcinoma in situ
ASCUS: Atypical squamous cells of undetermined significance
BMI: Body mass index
CI: Confidence interval
DNA: Dioxyribonucleic acid
dUTP/UNG: Uridine-5’-triphosphate/uracil-N-glycosylase
HPV: Human papillomavirus
HrHPV: High risk human papillomavirus
HSIL: High grade suqamous epithelial lesion
IARC: International agency for research on cancer
IBK: Innsbruck
ICO: Institut Catalana D‘Oncologia
IL: Innsbruckland
LSIL: Low grade squamous epithelial lesion
OR: Odds ratio
PCR: Polymerase chain reaction
SD: Standard deviation
SIR: Standardized incidence rate
WHO: World Health Organization
