# Supplementary figures and images for: Pre-vaccine era cervical human papillomavirus infection among screening population of women in west Austria
Source: BMC Public Health. 2016 Aug 26;16(1):889. doi: 10.1186/s12889-016-3581-0 (PMC5002092; doi:10.1186/s12889-016-3581-0)

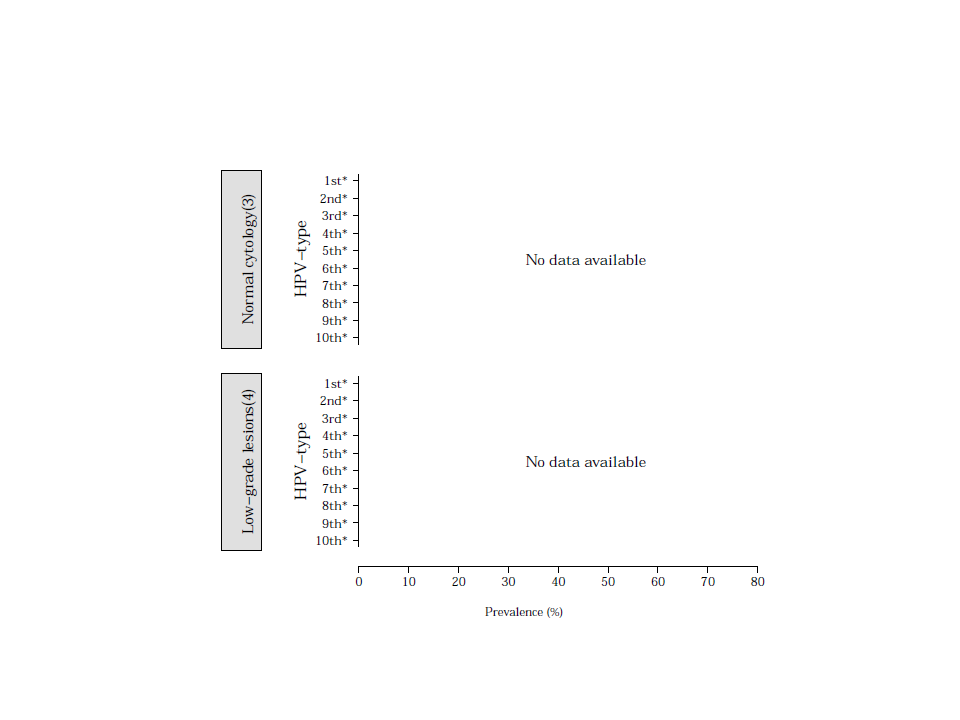

Supplement: Additional file 1: Figure S1. — Prevalence and distribution of ten most frequent HPV genotypes among women with no or low grade cervical lesions in Austria. (http://www.hpvcentre.net/statistics/reports/AUT_FS.pdf). Accessed March 2016. (TIF 48 kb) [file 12889_2016_3581_MOESM1_ESM.tif]

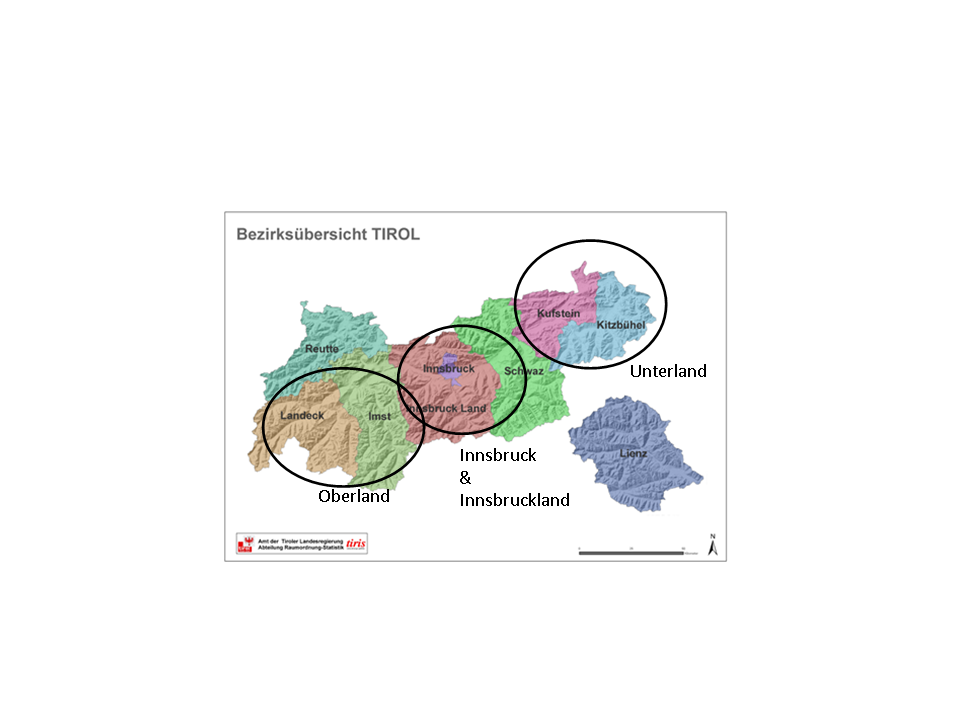

Supplement: Additional file 2: Figure S2. — Map of Tyrol, West Austria: Districts included in the study (circled) (Tyrolean regional government website. https://www.tirol.gv.at/fileadmin/themen/landesentwicklung/raumordnung/bilder/tiris/bezirksuebersicht_tirol_01.jpg. Accessed September 2015. (TIF 167 kb) [file 12889_2016_3581_MOESM2_ESM.tif]

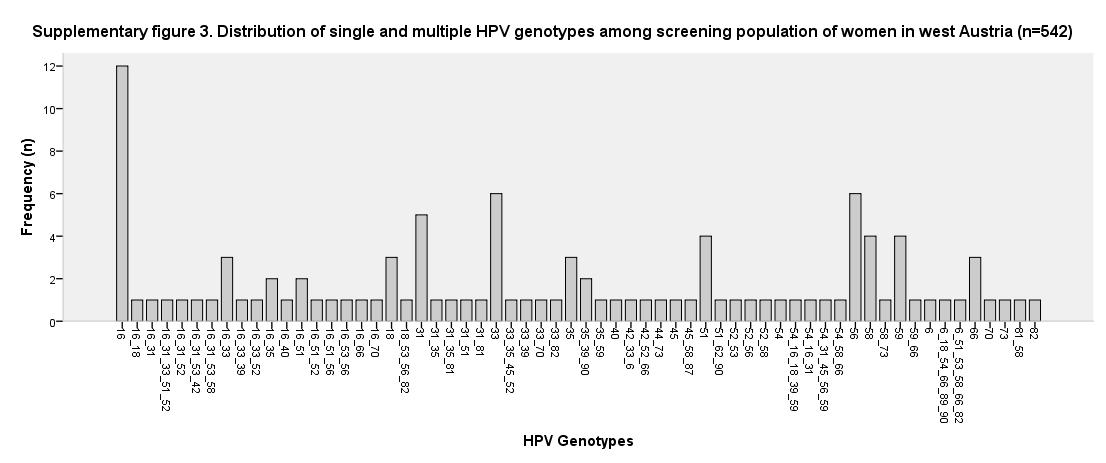

Supplement: Additional file 3: Figure S3. — Distribution of single and multiple HPV genotypes among screening population of women in west Austria (n=542). (JPG 52 kb) [file 12889_2016_3581_MOESM3_ESM.jpg]

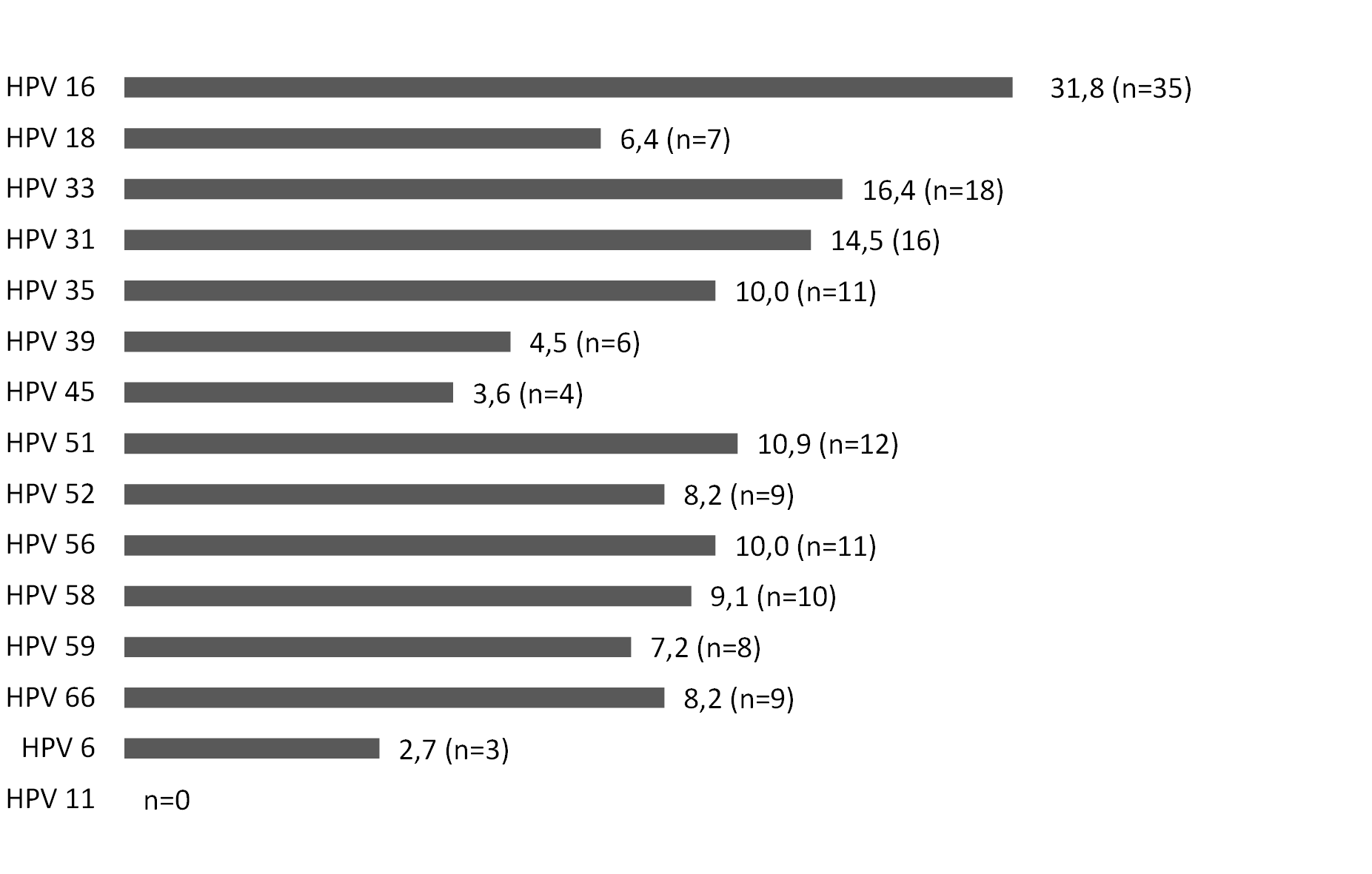

Supplement: Additional file 4: Figure S4. — Distribution (%) of HPV genotypes (high risk types, HPV 6 and HPV 11) among screening population of women in west Austria. (TIF 221 kb) [file 12889_2016_3581_MOESM4_ESM.tif]
